# Highlights in BACE1 Inhibitors for Alzheimer's Disease Treatment

**DOI:** 10.3389/fchem.2018.00178

**Published:** 2018-05-24

**Authors:** Judite R. M. Coimbra, Daniela F. F. Marques, Salete J. Baptista, Cláudia M. F. Pereira, Paula I. Moreira, Teresa C. P. Dinis, Armanda E. Santos, Jorge A. R. Salvador

**Affiliations:** ^1^Laboratory of Pharmaceutical Chemistry, Faculty of Pharmacy, University of Coimbra Coimbra, Portugal; ^2^Center for Neuroscience and Cell Biology, University of Coimbra Coimbra, Portugal; ^3^Chem4Pharma, Edifício IPN Incubadora Coimbra, Portugal; ^4^Faculty of Medicine, University of Coimbra Coimbra, Portugal; ^5^Laboratory of Physiology, Faculty of Medicine, University of Coimbra Coimbra, Portugal; ^6^Laboratory of Biochemistry, Faculty of Pharmacy, University of Coimbra Coimbra, Portugal

**Keywords:** Alzheimer's Disease (AD), amyloid-β (Aβ), BACE1, inhibitors, small molecules, drug discovery and development

## Abstract

Alzheimer's disease (AD) is a severe neurodegenerative disorder and the most common type of dementia in the elderly. The clinical symptoms of AD include a progressive loss of memory and impairment of cognitive functions interfering with daily life activities. The main neuropathological features consist in extracellular amyloid-β (Aβ) plaque deposition and intracellular Neurofibrillary tangles (NFTs) of hyperphosphorylated Tau. Understanding the pathophysiological mechanisms that underlie neurodegeneration in AD is essential for rational design of neuroprotective agents able to prevent disease progression. According to the “Amyloid Cascade Hypothesis” the critical molecular event in the pathogenesis of AD is the accumulation of Aβ neurotoxic oligomers. Since the proteolytic processing of Amyloid Precursor Protein (APP) by β-secretase (beta-site APP cleaving enzyme 1, BACE1) is the rate-limiting step in the production of Aβ, this enzyme is considered a major therapeutic target and BACE1 inhibitors have the potential to be disease-modifying drugs for AD treatment. Therefore, intensive efforts to discover and develop inhibitors that can reach the brain and effectively inhibit BACE1 have been pursued by several groups worldwide. The aim of this review is to highlight the progress in the discovery of potent and selective small molecule BACE1 inhibitors over the past decade.

## Introduction

Dementia has a tremendous emotional impact on the individual and his family, as well as an immense percussion at the economic level. Dementia was recognized by WHO in 2017 as a public health priority, thereafter a global intervention aiming to achieve physical, mental and social wellbeing for people with dementia, their caregivers and families, both for present and future generations, was planned (WHO, 2017).

Alzheimer's disease (AD), which accounts for 60–70% of all dementias, is clinically characterized by a gradual loss of memory and cognitive abilities, as well as by altered behavior, ultimately causing disability and dependency (Masters et al., 2015). Essential areas to cognitive processes in the brain are particularly damaged during the disease development, leading to early synapse loss, neuronal dysfunction, and death culminating in brain atrophy, which are responsible for the manifestation of the disease (Querfurth and LaFerla, 2010). An accumulation of misfolded proteins—amyloid-β (Aβ) and Tau protein—marks the AD brain. The “Amyloid Cascade Hypothesis” proposes that the cerebral appearance of aggregates of oligomeric Aβ plays a central role for AD neuropathogenicity (Karran et al., 2011; Selkoe and Hardy, 2016). These neurotoxic agents may interact with many neuronal receptors triggering a neurodegenerative cascade of events that are responsible for mitochondria dysfunction (Hauptmann et al., 2006; Carvalho et al., 2015), endoplasmic reticulum stress (Correia et al., 2015; Plácido et al., 2015), oxidative stress (Carvalho et al., 2016), DNA damage, neuroinflammation, among other deleterious mechanisms. The Aβ oligomers can also disrupt normal kinase and phosphatase activity, resulting in the hyperphosphorylation of Tau protein and subsequent Neurofibrillary tangles (NFTs) formation (Resende et al., 2008).

The current pharmacological therapeutics for AD only attenuate the symptoms, and do not affect the mechanisms underlying disease progression. Disease-modifying approaches have recently become the focus of AD research, aiming to prevent the neurodegenerative process at early phases before clinical manifestations (Bachurin et al., 2017; Cummings et al., 2017). The main directions include the search for drugs that are aimed at blocking the pathogenic steps responsible for the appearance of the pathological structures of AD—Aβ plaques and NTFs. Thus, several research groups are seeking to develop small molecules that can act as anti-amyloidogenic agents via inhibiting Aβ production and aggregation and promoting its removal (Kulshreshtha and Piplani, 2016).

The BACE1 is the transmembrane aspartyl protease that cleaves APP at the β-site. The sequential proteolytic cleavage of APP by BACE1 and γ-secretase leads to the production and release of Aβ peptide in the brain. Therefore, amyloidogenic secretases are key therapeutic targets being currently explored for AD-modifying intervention. Several studies report that BACE1 inhibitors hold great potential as a potential strategy in decreasing Aβ brain concentrations, thus preventing the progression of AD (Citron, 2002; De Strooper et al., 2010). Since BACE1 identification in 1999 (Vassar, 1999), inhibitors covering many different structural classes have been extensively reported in the literature (Ghosh et al., 2012; Ghosh and Osswald, 2014; Menting and Claassen, 2014; Yan and Vassar, 2014).

Here we will provide a summary overview of the recent advances in structural evolution of BACE1 inhibitor classes in order to improve their potency and selectivity.

## Challenges in BACE1 inhibitor development

BACE1 inhibitors can be identified into two main categories, peptidomimetics and nonpeptidics, and further subclassification can be made based on the core functional groups that interact with the catalytic dyad (Ghosh and Osswald, 2014). The development of OM99-2, an inhibitor based on the APP cleavage site sequence, demonstrated for the first time the druggability of BACE1. Therefore, the elucidation of the enzyme-ligand complex crystal structure gave much insight into the binding mode of inhibitors in the BACE1 binding pocket (Ghosh et al., 2001).

The development of effective BACE1 inhibitors faces many challenges. First, due to the location of BACE1 in the brain and in the lumen of endosomes, inhibitors need to cross the blood-brain barrier (BBB) and neuronal membranes to access the target (Ben Halima et al., 2016). Although peptidomimetic inhibitors are highly potent *in vitro*, their inherent poor drug properties (reduced brain permeability, short half-life and reduced oral availability) account for their low *in vivo* efficacy. In fact, one of the major issues in the design of recent generations of inhibitors is the transition from peptidomimetics to brain penetrant small molecules (Oehlrich et al., 2014).

Furthermore, concerning the BACE1 inhibitors' discovery process, there are some important structural features of the enzyme that should be wisely taken into consideration, namely the catalytic aspartic dyad, structural flexibility, and the large binding pocket (Dislich and Lichtenthaler, 2012; Yuan et al., 2013). Due to the large catalytic pocket of BACE1 the inhibitors are required to be large enough to interact within the active site, yet these inhibitors should be small enough to exhibit suitable drug-like properties (Vassar, 2016). Moreover, to prevent cross-inhibition toxic effects, it is especially important to ensure that BACE1 inhibitors are selective over other aspartic proteases because these enzymes are closely related to each other since the two catalytic aspartic acid residues are conserved across the class (Ghosh and Osswald, 2014). Additional challenges to overcome include the susceptibility to P-glycoprotein (P-gp) efflux and the inhibition of hERG (human Ether-A-Go-Go ion channel) related to cardiotoxicity, as well as the inhibition of cytochrome enzymes to avoid drug interactions by BACE1 inhibitors (Brodney et al., 2015). Beyond APP processing, BACE1 has several cellular substrates and its physiological role is required for optimal cognitive function. The BACE1-null mice present neuronal phenotypes including synaptic dysfunction, retinopathy, epileptic seizures, hypomyelination, etc., that appear to be related to the abolished cleavage of BACE1 substrates. Therefore, BACE1 inhibitors might exhibit mechanism-based toxicity (Barão et al., 2016). Although more studies are needed to further understand BACE1 biological functions and the consequences of its chronic inhibition, a careful titration of drug dosage should be considered when using BACE1 inhibitors.

Hence, the focus has been searching for inhibitors that can effectively and selectively inhibit BACE1 *in vivo*. Some of them have shown benefits in preclinical studies and six drugs, JNJ-54861911, CNP520, LY3202626, Elenbecestat, Lanabecestat, and Verubecestat, are currently being tested in human clinical trials on different types of populations, including patients with mild-to-moderate AD and in patients at risk for AD (Yan, 2016; Mullard, 2017). Verubecestat was the first inhibitor to progress to clinical phase 3 and the best positioned drug to confirm the safety and efficacy of BACE1 inhibition. However, Merck has halted its pivotal trial in patients with mild-to-moderate AD and the trial in patients with prodromal AD due to ineffectiveness in cognitive decline reduction and unlikely positive benefit/risk outcome, respectively (Staff, 2017; Merck, 2018). Consequently, these unfortunate drawback raises questions about the clinical use of BACE1 inhibitors as potential anti-AD drugs.

One the other hand, recent evidence obtained in an animal model where deletion of BACE1 was performed at the adult stage to mimic BACE1 inhibition in AD patients, demonstrates that sequential and gradual BACE1 inhibition can completely reverse amyloid pathology. However, caution is needed in translating these findings to the clinical practice since BACE1 is required for cognitive functions and BACE1 inhibitors are expected to be used for chronic treatment (Hu et al., 2018). Another recent study, using a longitudinal approach with two-photon microscopy to monitor the impact of pharmacological BACE1 inhibition on early Aβ plaque deposition in an AD mouse model, demonstrated that it slows down progression of initial Aβ plaque formation but was less effective toward existing plaques (Peters et al., 2018). Therefore, the timing of intervention with BACE1 inhibitors in clinical trials may be adjusted according to the emerging data to achieve optimal therapeutic efficacy for AD treatment.

In addition, a diversity of complex medicinal chemistry approaches, such as high throughput and fragment-based screening, X-ray crystallography, nuclear magnetic resonance (NMR) and computational methods, have been directed in recent years toward the development of a wide variety of scaffolds of small BACE1 inhibitors. Also, the knowledge of BACE1 structure has helped to conduct a structure-based (SB) design of novel BACE1 inhibitors, and promising findings have been obtained, some of them described herein.

### Amino/iminohydantoins

Small molecule derivatives with aminohydantoin scaffold have been reported as BACE1 inhibitors, including the disubstituted pyridinyl aminohydantoins with low nanomolar potency and high selectivity for BACE1 (Malamas et al., 2010a,b,c, 2011; Zhou et al., 2010) and the small and rigid spirocyclic aminohydantoin analogs, which were designed through a structure-activity relationship (SAR) approach to improve their *in vivo* activity (Hunt et al., 2013). Furthermore, derivatives of 7-tetrahydropyran-2-yl chromans and 8-tetrahydropyran-2-yl chromans were also prepared. Using structure-based design these chemotypes were substituted with three different aspartate-binding groups: aminohydantoin, aminooxazolines, and aminothiazolines, in an attempt to modulate potency, selectivity, efflux, and permeability. In the first chemotype, the aminohydantoin analogs were the most potent and selective against Cathepsin-D (Thomas et al., 2014a,b).

The potent and brain penetrant class of iminohydantoin BACE1 inhibitor was revealed via a fragment-based NMR screening followed by X-ray crystallography (Zhu et al., 2010). Later, a new set of iminohydantoin analogs identified through a SB approach, showed selectivity and high-affinity for BACE1, and a low level of central Aβ after oral administration in rats (Cumming et al., 2012). More recently, iminohydantoin spiropiperidine methyl-substituted derivatives were identified by molecular modeling methods used to explore available binding pockets adjacent to the ligand binding. As a result, the addition of a placed methyl group maintained the required pKa of the piperidine nitrogen and occupied a small hydrophobic pocket, stabilizing the ligand into the active site. Compound **1** (listed in Table 1) showed an improvement in BACE1 inhibitory potency and good selectivity against Cathepsin-D (Egbertson et al., 2015).

**Table 1:** Representative structures of potent and selective BACE1 inhibitors scaffolds.

**Scaffold**	***In vitro* activity**	**References**	**Scaffold**	***In vitro* activity**	**References**
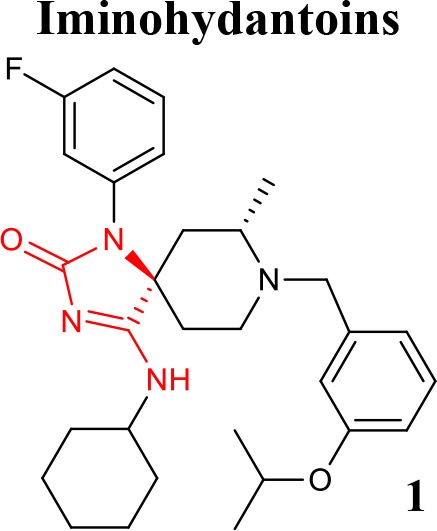	BACE1 IC_50_ = 6 nM HEK293 Cell sAPPβ IC_50_ = 63 nM BACE2 IC_50_ = 2 nM CatD IC_50_ = 16.3 μM	Egbertson et al., 2015	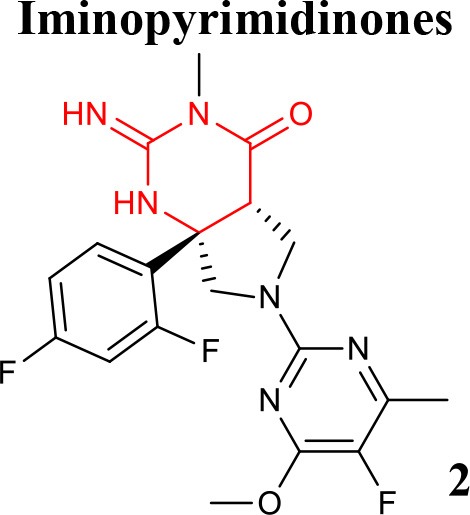	BACE1 *K*i = 5 nM HEK293 Cell Aβ_40_ IC_50_ = 14 nM CatD/BACE1 > 458 hERG IC_50_ = 1.9 μM	Mandal et al., 2016
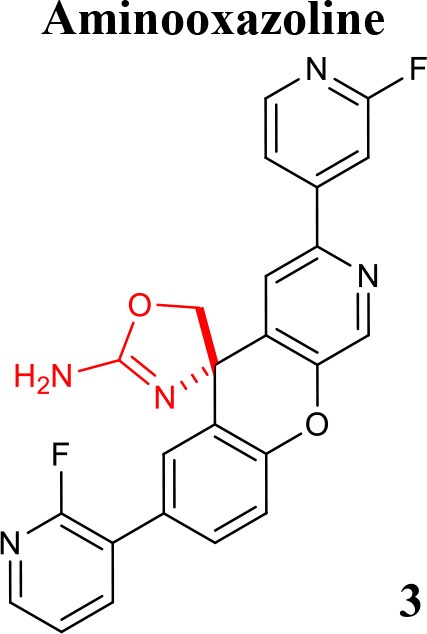	BACE1 IC_50_ = 0.9 nM HEK293 Cell IC_50_ = 21.2 nM CatD IC_50_ = 0.66 μM hERG *K*i >10 μM	Chen et al., 2015	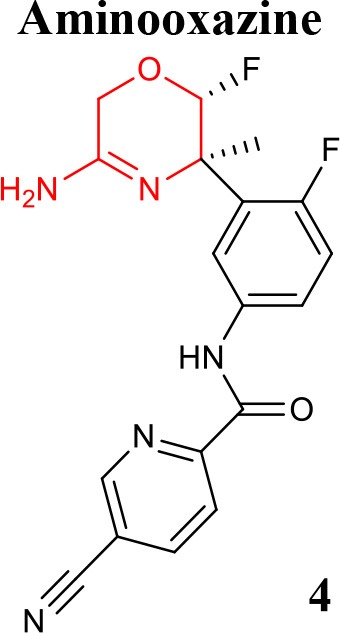	BACE1 IC_50_ = 7.6 nM SKNBE2 Cell Aβ_42_ IC_50_ = 8.1 nM hERG %inhibition at 3 μM = 56%	Rombouts et al., 2015
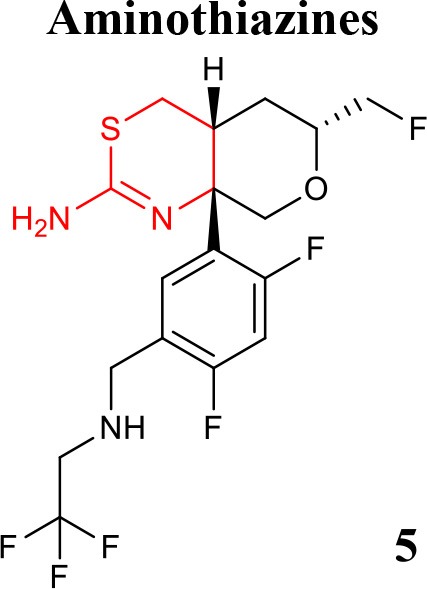	BACE1 IC_50_ = 77 nM H4 Cell sAPPβ IC_50_ = 0.006 nM BACE2 IC_50_ = 0.295 μM CatD IC_50_ > 100 μM CYP2D6 IC_50_ = 9.1 μM hERG IC_50_ = 4.3 μM	Butler et al., 2017	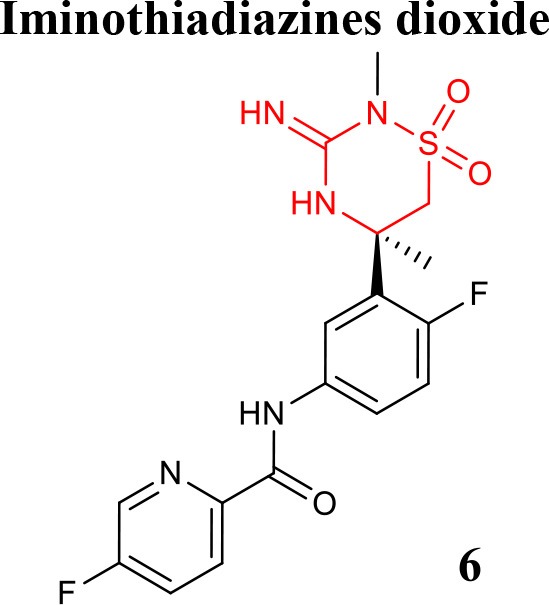	BACE1 *K*i = 2.2 nM HEK293 Cell Aβ_42_ IC_50_ = 0.7 nM BACE2 *K*i = 0.38 nM CatD *K*i >>100,000 nM hERG IC_50_ = 2.2 μM	Scott et al., 2016
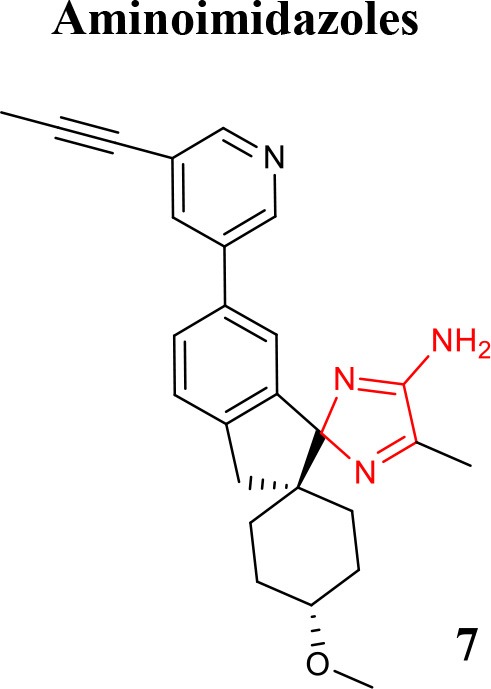	BACE1 *K*i = 0.4 nM BACE1 IC_50_ = 0.6 nM SH-SY5Y Aβ_40_ Cell IC_50_ = 0.08 nM BACE2 IC_50_ = 0.9 nM CatD IC_50_ = 16.1 μM hERG IC_50_ = 4.7 μM	Eketjäll et al., 2016	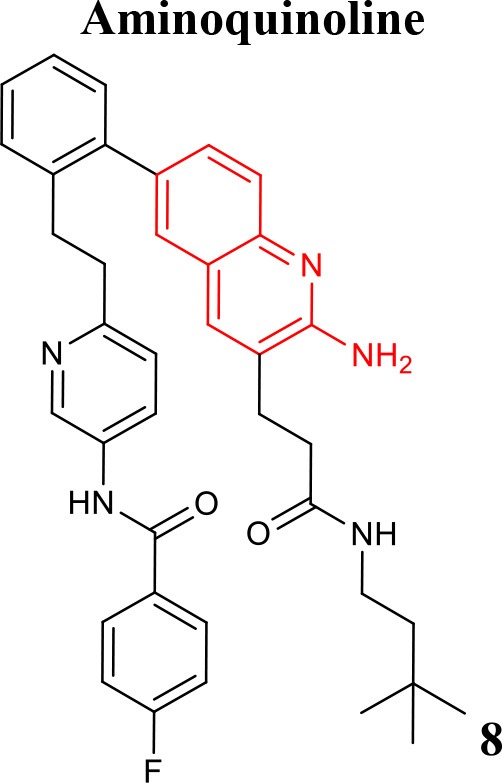	BACE1 IC_50_ = 0.8 nM CatD IC_50_ = 1.9 μM	Jordan et al., 2016
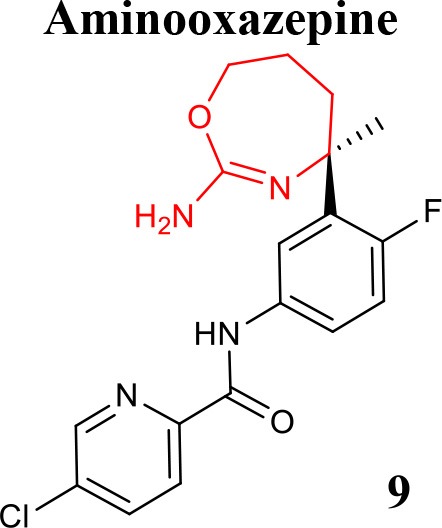	BACE1 IC_50_ = 14 nM HEK293 Cell Aβ_40_ IC_50_ = 11 nM	Woltering et al., 2013	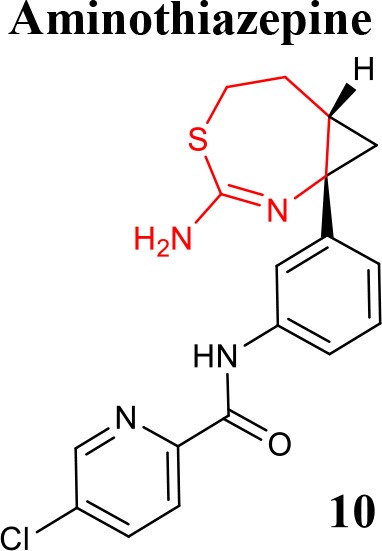	BACE1 IC_50_ = 27 nM HEK293 Cell Aβ_40_ IC_50_ = 2 nM	Woltering et al., 2013

### Iminopyrimidinones

A new class of orally bioavailable and BBB permeable BACE1 inhibitors with iminopyrimidinone scaffold was obtained by SB optimization of iminohydantoin chemotype. The best compound of these series potently reduced Aβ levels when administered orally to rats (Stamford et al., 2012). Mandal and collaborators have also identified potent BACE1 inhibitors with iminopyrimidinone core (Mandal et al., 2012). Thus, seeking to identify novel, highly soluble, permeable, and selective inhibitors minimizing off-target liabilities, a set of fused pyrrolidine iminopyrimidinone derivatives were recently prepared using a SB approach. As a result of these successful efforts, the difluorophenyl compound **2** (listed in Table 1), which represents a novel molecular shape, was identified. This compound has been shown to strongly lower central Aβ levels following oral administration of a single dose in preclinical assays and to display good selectivity over Cathepsin-D, without significant inhibitory effect toward a range of human cytochrome enzymes, thus having minimal risk of interaction with other drugs (Mandal et al., 2016).

### Aminooxazolines and aminooxazines

Over the last years, a variety of aminooxazoline derivatives, including a class of aminooxazoline xanthenes have been reported as BACE1 inhibitors (Huang et al., 2012; Epstein et al., 2014). Moreover, a class of aminooxazoline 4-aza substituted on the xanthene core shows good BACE1 potency while reducing hERG binding affinity (Dineen et al., 2014). A further optimization based on SB design with balance of physicochemical parameters conducted to 3-aza-4-fluoroxanthene analogs. It was found that the incorporation of a nitrogen atom into the 3-position of the xanthene scaffold improved BACE1 potency, reduced hERG binding affinity and improved cell-permeability (Cheng et al., 2015). Further efforts though SAR generated several potent and orally active BACE1 inhibitors. The compound **3** (listed in Table 1) was identified as the most attractive candidate within this series, exhibiting low *in vivo* clearance, good oral bioavailability, and reduced potential for cardiovascular liabilities in preclinical species (Chen et al., 2015).

Efforts to obtain fluorine-containing aminooxazines derivatives resulted in a class characterized by high *in vivo* activity and good drug properties. Through an extensive fluorine scan study it was revealed the impact of fluorine to lower the pKa and to change the pharmacological profile of this class of BACE1 inhibitors (Hilpert et al., 2013). Recently, the effort to reduce the amidine pKa while optimizing interactions with the BACE1 active site, allowed to modulate cell-permeation ability and P-gp efflux. This enabled the discovery of orally bioavailable and centrally active 1,4-oxazines derivatives that robustly lowered brain Aβ levels. Compound **4** (listed in Table 1) was identified as the most promising within this series (Rombouts et al., 2015).

### Aminothiazines and iminothiadiazines dioxide

Two potent aminothiazine BACE1 inhibitors developed by Eli Lilly, LY2811376, and LY2886721, advanced into the clinics with a good pharmacokinetic profile. Unfortunately, these inhibitors have been discontinued due to preclinical retinal toxicity and to clinical abnormal liver biochemistry, respectively (May et al., 2011, 2015).

Recently, a lot of strategies have been implemented to disclose novel and potent aminothiazine BACE1 inhibitors, namely the truncation of the S3 substituent of the biaryl aminothiazine, which enables oral bioavailability and brain penetration (Wu et al., 2016b), and the preparation of fused bicyclic series of furo[2,3-*d*][1,3]thiazinamine derivatives (Wu et al., 2016a). Additional series of truncated and fused thioamidines were reported as efficiently selective in inhibiting BACE1 and with suitable pharmacokinetic profile (Butler et al., 2015). Further development guided by the crystal structures of CYP2D6 and BACE1 complexed with inhibitor and its corresponding metabolic product, led to improved potency, central efficacy, low clearance, and reduced hERG activity and risk of drug-drug interactions (Brodney et al., 2015). To this end, new derivatives with an aminomethyl linker that engages Gly230, a key residue in the BACE1 binding pocket and display high potency and improved brain penetration were disclosed. Therefore, a strategy that was implemented to prevent the drug-drug interactions predicted with basis on CYP2D6 binding affinities, led to the design of compound **5** (listed in Table 1), which exhibits robust *in vivo* efficacy (Butler et al., 2017).

Iminothiadiazine dioxides core derivatives have revealed high inhibitory activity against BACE1. Efforts have culminated in the discovery of the clinical Verubecestat (compound **6** listed in Table 1) that significantly lowered central Aβ levels in preclinical and in humans after oral administration. Despite not being selective over BACE2, it has highly selectivity compared to Cathepsin-D (Scott et al., 2016).

### Aminoimidazole and aminoquinoline

Structures with high potency and selectivity for BACE1 with aminoimidazole scaffold were firstly reported in 2009 (Malamas et al., 2009). The effort to improve cell-permeability and to reduce efflux properties in a new series of bicyclic aminoimidazoles BACE1 inhibitors was described to achieve *in vivo* brain efficacy (Swahn et al., 2012). The design of new amino-2*H*-imidazoles derivatives was performed to obtain new inhibitors with high activity, good drug properties, and low effect on hERG in combination with great *in vivo* exposure (Gravenfors et al., 2012). The clinical BACE1 inhibitor Lanabecestat developed by AstraZeneca (compound **7** listed in Table 1) belongs to the aminoimidazole class and is characterized as potent, highly permeable, orally active, BBB-penetrant with a very slow off-rate from BACE1, resulting in prolongation of the observed effect (Eketjäll et al., 2016).

A library of potent BACE1 inhibitors with 2-aminoquinoline core was designed through a complex strategy, including fragment-based screening and further SAR development supported by X-ray crystallography and molecular modeling studies (Cheng et al., 2011). In an effort to improve potency and selectivity of this class of compounds a fragment-linking approach using 19F NMR spectroscopy was conducted. This technique revealed to be useful in the identification of a second-site fragment that binds to a specific pocket of BACE1, which ultimately increased the potency while maintaining reasonable ligand efficiency with improved selectivity over Cathepsin-D. Compound **8** (listed in Table 1) exhibited a high-fold gain in its affinity and in selectivity for BACE1 (Jordan et al., 2016).

### Aminooxazepines and aminothiazepines

A series of seven-membered aminooxazepine and aminothiazepine derivatives was described as BACE1 inhibitors (Blass, 2012) and the effect of physicochemical properties, basicity and P-gp efflux on their activity was evaluated. It was demonstrated that compounds less basic, P-gp free and more lipophilic can achieve a high exposure profile. The 2-aminooxazepine derivatives were identified as promising brain penetrants, and compound **9** (listed in Table 1) showed high BACE1 inhibitory potency. Moreover, a series of cyclopropyl-fused oxazepine and thiazepine analogs was also prepared (Blass, 2013). The fusion of the cyclopropyl was shown to lower pKa of the functional group and the orientation of the ligand into the binding pocket. The cyclopropyl-fused 1,3-thiazepine compound **10** (listed in Table 1) is highly active in *in vitro* enzymatic and cellular studies and more potent than the oxygen analog cyclopropyl-fused-1,4-homo-oxazepine (Woltering et al., 2013).

### Recent structures reported as BACE1 inhibitors

Recently, the identification of new structural classes of compounds with BACE1 inhibitory activity at micromolar level via *in silico* methods was reported (listed in Table 2). Jain and colleagues identified derivatives with sulfonyl-amino-acetamide and alkylidene hydrazinecarboximidamide cores acting on BACE1. Compounds **11** and **12** were demonstrated as the most active of each chemotype, respectively (Jain et al., 2016a,b). De Tran and collaborators reported (3S,4S)-4-aminopyrrolidine-3-ol derivatives as potent BACE1 inhibitors and selective over BACE2 and Cathepsin-D. Compound **13** was identified as the most promising derivative (De Tran et al., 2016). In 2017, several new chemical structures acting as BACE1 inhibitors were described, among them—quinazolinone-based hydrazone derivative 14 (Haghighijoo et al., 2017), 2-substituted-thio-N-(4-substituted-thiazol/1*H*-imidazol-2-yl)acetamide derivative 15 (Yan et al., 2017), imidazopyridine derivative 16 (Azimi et al., 2017) and 2-oxopiperazine derivative 17 (Ghosh et al., 2017), were those that showed the best BACE1 inhibitory activity. These findings can be promising for further advance in the field of BACE1 inhibitors.

**Table 2:** Recent reported structures with BACE1 inhibitory activity.

**Scaffold**	***In vitro* activity**	**Reference**	**Scaffold**	***In vitro* activity**	**Reference**
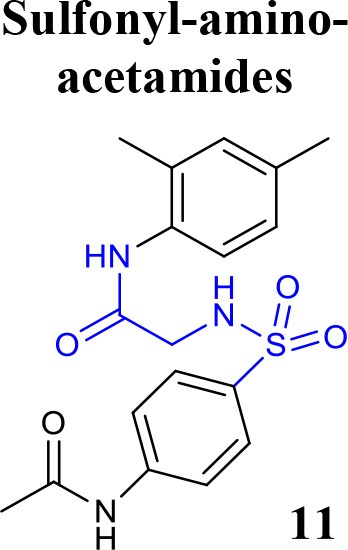	BACE1 IC_50_ = 7.90 μM %inhibition BACE1 at 10 μM = 61.90%	Jain et al., 2016a	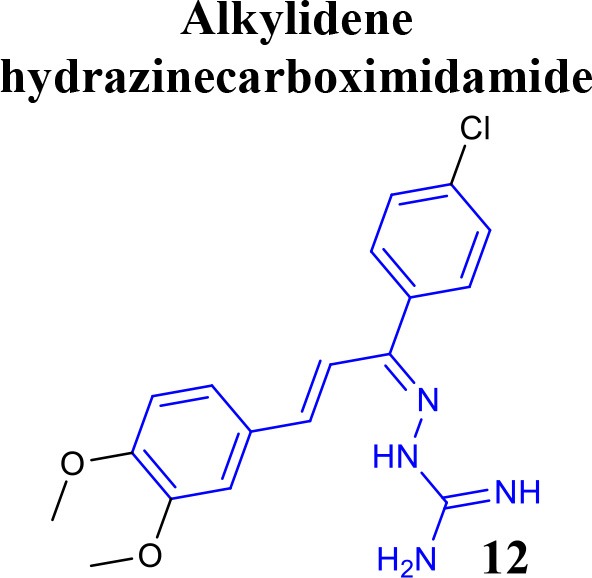	BACE1 IC_50_ = 6.42 μM %inhibition BACE1 at 10 μM = 78.23%	Jain et al., 2016b
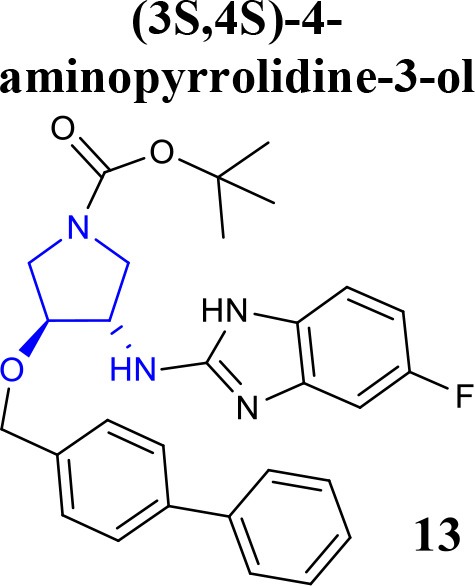	BACE1 IC_50_ = 0.12 μM PC12-APP_SW_ Cell IC_50_ = 1.7 μM BACE2 IC_50_ = 8.9 μM %inhibition CatD at 10 μM = 5.7% CatD IC_50_ >> 10 μM	De Tran et al., 2016	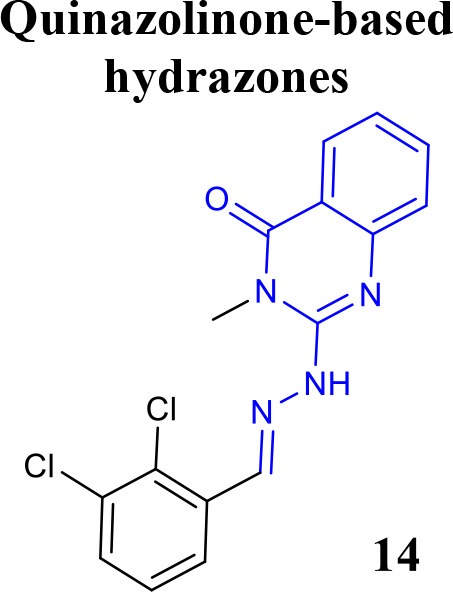	BACE1 IC_50_ = 3.7 μM %inhibition BACE1 at 10 μM = 66.0%	Haghighijoo et al., 2017
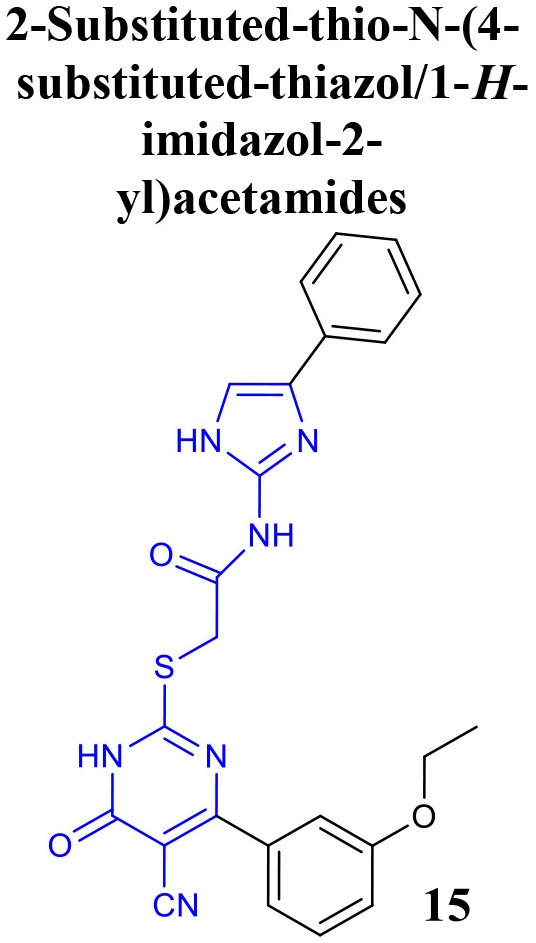	BACE1 IC_50_ = 4.6 μM %inhibition BACE1 at 10 μM = 84.5 %	Yan et al., 2017	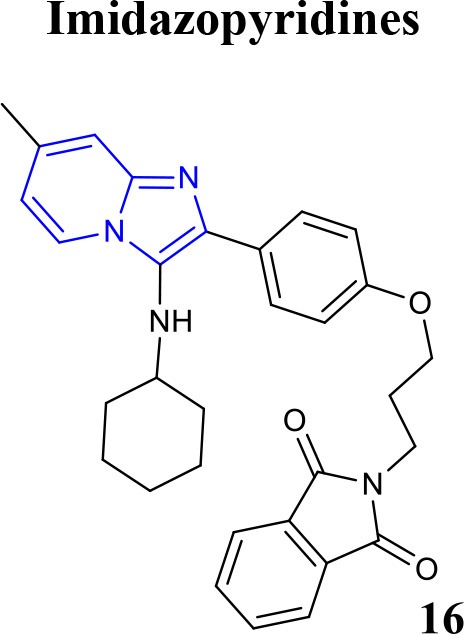	BACE1 *K*i = 4.38 nM BACE1 IC_50_ = 2.84 μM %inhibition BACE1 at 10 μM = 61.32%	Azimi et al., 2017
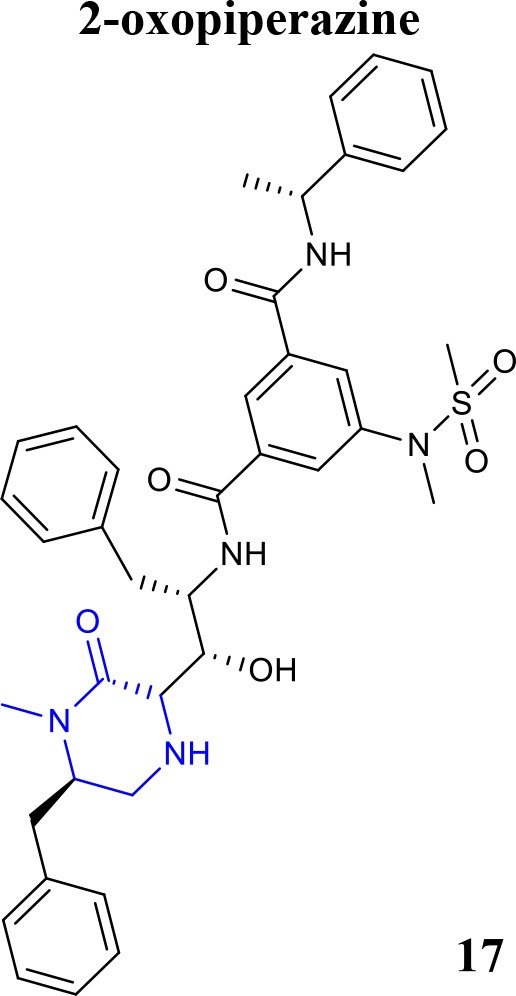	BACE1 *K*i = 12 nM	Ghosh et al., 2017			

## Future perspectives

The therapeutic potential of BACE1 inhibition has been investigated in the last decade. However, even though inhibitors efficiently lower brain Aβ levels, clinical trials still do not demonstrate improvements in cognitive function when administered to mild-to-moderate AD patients, calling into question the real worth of these potential anti-AD drugs and the clinical trials design. Emerging evidences suggest that the optimal timing for treatment with BACE1 inhibitors should be as early as possible. The optimal time point for a treatment with BACE1 inhibitors is a crucial issue that possibly explains some of the previous failures. The advances in technological diagnostic tools allowing better identification of patients at risk of developing AD, will enable clinical trials at preclinical stage when BACE1 inhibitors are expected to have the largest impact on amyloid plaques (Voytyuk et al., 2018).

Moreover, taking into account the complexity of AD, recent studies report multitarget approaches centered on BACE1, whose ligands are developed as small molecules that can modulate both BACE1 and other AD-related targets within synergistically pathways (Prati et al., 2018). Noteworthy, as BACE1 biological functions may be affected upon inhibition, further studies are required to develop efficient strategies to minimize the side-effects associated with long-term use of BACE1 inhibitors (Yan, 2017).

Indeed, although design and development of BACE1 inhibitors have proved extremely challenging, there is a substantial body of evidence that BACE1 inhibitors can act as disease-modifying agents and their use should be pursued to achieve benefits for AD patients.

## Author contributions

JC, DM, SB, CP, PM, and TD drafted the work and wrote the manuscript. AS and JS revised the manuscript. All authors approved the manuscript in its final form for publication.

### Conflict of interest statement

The authors declare that the research was conducted in the absence of any commercial or financial relationships that could be construed as a potential conflict of interest. The reviewer, MB, and handling Editor declared their shared affiliation.
